# Childhood adverse events and *BDNF* promoter methylation in later-life

**DOI:** 10.3389/fpsyt.2023.1108485

**Published:** 2023-02-24

**Authors:** Aoshuang Zhou, Marie-Laure Ancelin, Karen Ritchie, Joanne Ryan

**Affiliations:** ^1^Division of Epidemiology, Jockey Club School of Public Health and Primary Care, Chinese University of Hong Kong, Hong Kong, Hong Kong SAR, China; ^2^Institute for Neurosciences of Montpellier, University of Montpellier, INSERM, Montpellier, France; ^3^School of Public Health and Preventive Medicine, Monash University, Melbourne, VIC, Australia

**Keywords:** *BDNF*, early-life adversities, stress, blood, DNA methylation, epigenetics, older adults

## Abstract

Studies have shown that the effects of early-life stress and trauma can be enduring, with long-term negative effects on health. Epigenetics, including DNA methylation, have been implicated as a potential mechanism for these effects. Brain-derived neurotropic factor (BDNF) is a neurotransmitter involved in learning and memory, and altered *BDNF* promoter methylation measured in peripheral tissue has been found with early-life stress. However, whether such methylation differences remain stable into later life, is unknown. This study aimed to investigate the association between childhood adversity and *BDNF* promoter methylation in adults aged 65 years and over. Data came from a large study of older community-dwelling individuals in France (ESPRIT). Information on three major childhood adverse events, namely abuse/maltreatment, war/natural disaster, and financial difficulties/poverty, was obtained by retrospective reporting from participants of ESPRIT study. *BDNF* promoter I and IV methylation was assessed in blood and buccal tissue. Linear regression analysis was performed, adjusting for age, sex, education, depression, and morbidity. Among 927 participants, there was no strong evidence that childhood abuse/maltreatment or financial difficulties/poverty were associated with *BDNF* methylation in older individuals. For war/natural disaster, differential methylation at four of twenty-nine CpG sites was observed, however, these would not have remained significant after correction for multiple testing. Together, these findings do not support a long-term association between adverse childhood events and *BDNF* methylation in older age, but further large prospective studies are needed, which do not target specific genes, but consider DNA methylation across the genome.

## Introduction

There is a well-established link between early-life adversity and poorer health outcomes. Individuals exposed to childhood adverse events are at higher risk of negative physical, mental, and behavioural-related health outcomes, in comparison to those without adversity (1–3). In addition, evidence suggests certain types of early-life adverse events may have different impacts. For example, while childhood abuse was found to be strongly associated with depression, the experience of family violence in childhood was found to be an important risk factor for the development of anxiety disorder (4). Furthermore, the associations may vary at different ages. Financial difficulties in childhood for example, may be associated with poorer mental health in adulthood, but not in older age (5). Childhood adversities can lead to acute and/or chronic lasting stress, which has been shown capable of influencing the programming of early-life stress-response system (6, 7) and brain development (8), with the potential for biological embedding and thus longer-term effects (9).

One promising mechanism underlying the long-term effects of early-life stress is epigenetic mechanisms. It refers to changes in DNA function that affect gene expression and protein synthesis, but without involving mutations in DNA sequence (10, 11). Three types of epigenetic mechanisms, including DNA methylation, histone modification, and microRNA-mediated processes, are of primary interest in research. DNA methylation is one of the most extensively studied epigenetic modifications, in which a methyl group is covalently bound to cytosine-phosphate-guanine (CpG) and may lead to the reduction of gene expression (12). Moreover, DNA methylation has been shown to be highly sensitive to environmental stimuli, especially in the critical early-life period, as this special time is essential for the development of neural plasticity and potential epigenetic marks may contribute to the change of vital physiological and neuroendocrine systems (13, 14). Studies have shown the adverse exposures early in life can result in changes to DNA methylation patterns which then remain stable over time (15, 16).

The brain-derived neurotrophic factor (BDNF), is a protein that is essential in neuronal development and function, as well as synaptic plasticity (17). BDNF levels have been shown to be highly dynamic in response to stress (18), and interacts directly with the hypothalamic-pituitary-adrenal (HPA) axis in regulating the stress response (19). Numerous studies have identified an association between psychiatric disorders, in particular depression, and altered *BDNF* promoter methylation (20–22). There is also some evidence to indicate differential *BDNF* promoter methylation following previous stress-related events. For example, maternal stress during pregnancy has been associated with differential *BDNF* methylation in the offspring (23, 24), as has neighbourhood disadvantage in early-life (25, 26). Furthermore, rodent models suggest early-life adversity alters *BDNF* methylation and persists into adulthood (27–29), and this was supported by a study of 85 human adults where differential blood *BDNF* methylation was observed in response to low levels of maternal care in childhood (6). To date, however, no studies have looked at the long-term effects of childhood adverse events on differential *BDNF* methylation in older adult populations.

Therefore, this study aimed to investigate whether major adverse events in childhood are associated with altered *BDNF* methylation in later life, investigating DNA methylation in both blood and buccal tissue.

## Methods

### Participants

This study involved participants from the ESPRIT cohort, an ongoing longitudinal study that aimed to investigate the prevalence of and risk factors for psychiatric disorders in French older adults aged at least 65 years (30). Participants were randomly selected from the electoral rolls and invited to participate if they met the eligible criteria during March 1999 and February 2001. In cases where an individual refused to participate, a second individual was randomly drawn from the same electoral division to ensure a representative sample. For inclusion they needed to be community-dwelling residents living in the Montpellier region in the south of France. Although participants with dementia were not excluded from ESPRIT, they were not invited to provide buccal samples and were not included in the current study. Written informed consent was obtained for all investigations, and ethical approval was given by the Ethical Committee of the University Hospital of Kremlin-Bicêtre, France.

Participants were followed up at a two-year interval. At baseline and each follow-up, a half-day assessment, including a standardised health interview, neurological examination, and psychiatric interview, was conducted with the assistance of a neurologist and an interviewer (psychologist or nurse).

### Assessment of childhood adverse events

A retrospective self-report questionnaire containing 25 adverse experiences during childhood was developed based on existing validated instruments, with full details described in a previous publication (31). It was administered four years after recruitment, at the third wave of the study. The timing of the questionnaire was important to enable study interviewers sufficient time to establish close relationships with the participants and to facilitate the acquirement of sensitive information (31). The majority of the questions had a yes/no response option. Individuals were allowed to ask questions and to discuss the content of the questionnaire with interviewers.

We focused on three major childhood experiences which are likely to be considered as major stressful events that could directly impact the individual, and that were sufficiently common (e.g., reported by more than 20 individuals) to enable analyses. These were: early-life abuse or maltreatment (which included five individual items: neglect; verbal abuse from their parents; humiliation, harassment or mental cruelty; physical and/or sexual abuse; excessive physical punishment for misbehavior), experiencing a war event or natural disaster, or poverty or extreme financial difficulties in childhood. These were chosen given our *prior* hypothesis that major stress can result in long-term effects on DNA methylation, and thus help explain associations with later health events. Indeed, in ESPRIT, we have already shown that all events were associated with an increased risk of late-life depression (31). Additionally, abuse/maltreatment and war/natural disaster were associated with an increased risk of mortality in later-life (32); while abuse/maltreatment and poverty was associated with poorer cognitive function (33).

### *BDNF* promoter DNA methylation analysis

Promoters I and IV were chosen as our primary interests, because previous research has shown that differential methylation in these regions is associated with multiple mental health issues (7, 34–37). Participants provided blood samples at baseline/study entry, and buccal swabs were collected at approximately the fourth wave of follow-up. DNA extraction kits from Amersham-Pharmacia Biotech were used to extract genomic DNA from white blood cells yielded from 15 ml EDTA (31), while genomic DNA of buccal swabs was extracted by salting out. Bisulphite conversion of DNA was performed using the EZ-96 DNA Methylation-Lightning MagPrep kit (Irvine, USA), and DNA was subsequently amplified in triplicate.

Two assays targeting the region of *BDNF* promoters I and IV were designed using the Agena Bioscience Epidesigner software.^1^ As described in detail previously (38, 39), these covered promoter I (chr11:27,744,025–27,744,278) and promoter IV (chr11:27,723,096–27,723,467) of the gene (locations given are on the UCSC h19 assembly). For promoter I, 11 CpG units were measured, and 7 CpG units across promoter IV, were investigated. DNA methylation data was obtained using Sequenom MassARRAY (San Diego, CA, USA) (40) and methylation ratios were calculated by EpiTyper software (Sequenom, San Diego, CA, v.1.2). In the case of multiple individual sites sharing a single methylation unit, the average methylation was provided. The average methylation level for samples with technical triplicate values within 10% of the median were included in analysis.

### Other characteristics

Sociodemographic, lifestyle, medical history, and psychological health information was collected at baseline. Participants were classified as overweight or obesity if body mass index (BMI) was between 25 and 30 or over 30 kg/m^2^, respectively. Smoking status was categorized as either ever smoking (>1 pack year) or not smoking (<1 pack year). Alcohol consumption was classed as high if they had an average of more than 24 g per day. Education level was dichotomized as whether at least secondary school was completed or not. The Mini International Neuropsychiatric Interview (MINI, French version 5.00) was used to diagnose current major depressive disorder, according to DSM-IV criteria, with detailed process being described elsewhere (30). The Center for Epidemiologic Studies Depression (CES-D) scale was used to assess the severity of depressive symptoms. Participants were defined as having depression if they had a CES-D score greater than 16, which is considered as a threshold of moderate to severe depression, or were diagnosed of current major depressive disorder. Global cognitive function was assessed by the MMSE (Mini-Mental State Examination), with scores greater than 26 being classified as not having cognitive impairments. A standardized interview was also conducted to obtain information on cardiovascular ischemic pathologies and chronic illness morbidity information.

### Statistical analysis

The univariate association between the three major adverse childhood experiences and *BDNF* methylation in later life was determined using *t*-tests, with raw *BDNF* methylation log transformed (natural log) to normalise the data. For nominally significant associations at a conservation *p*-value of <0.10, linear regression was then used to determine the associations adjusting for age, sex, education, depression, and morbidity. This higher cut-off for the *p*-value was chosen at this stage, to ensure that all associations which were potentially significant (*p* < 0.05) after adjustment for covariates were included. Additional adjustment for other covariates as described in Table 1, was also determined. 95% confidence interval was calculated and *p*-value < 0.05 was considered as significant. Stata version 16 (StataCorp, TX) was used to all analyses.

**TABLE 1:** Participant characteristics at baseline (*n* = 927).

	Characteristics	Mean (SD)	% (*n*)
Sociodemographic	Age	71.6 (4.5)	–
	Female gender	–	58.8 (544)
	Completed at least secondary school education	–	39.4 (365)
	BMI ≤25 kg/m^2^	–	57.5 (531)
Lifestyle	Overweight (BMI: 25–30 kg/m^2^)	–	35.2 (325)
	Obesity (BMI ≥30 kg/m^2^)	–	7.4 (68)
	Living alone	–	24.3 (225)
Physical health	High alcohol consumption (≥24 g per day)	–	19.0 (172)
	Ever smoking (>1 pack year)	–	40.0 (366)
	Chronic illness morbidity^a^	–	15.2 (141)
Psychological health	Cardiovascular ischemic pathologies^b^	–	10.5 (97)
	Depression^c^	–	22.9 (210)
	Lower MMSE, ≤26	–	76.4 (705)

^a^Chronic illness defined as at least one of the following: asthma, hypercholesterolemia (total cholesterol ≥6.2 mmol/L), hypertension (resting blood pressure ≥160/95 mmHg or treated), thyroid problems, cancer diagnosis in last 2 years, diabetes or fasting glucose ≥7.0 mmol/L or reported treatment.

^b^History of cardiovascular disease (for example, angina pectoris, myocardial infarction, stroke, cardiovascular surgery, arteritis, and vascular disease).

^c^Diagnosed according to DSM-IV criteria using the Mini-International Neuropsychiatric Interview (MINI) French Version 5.00.

## Results

### Study population

The study included 927 participants who had responded to the questionnaire on childhood adverse events, have DNA samples that could be used for analysis of *BDNF* methylation (i.e., provided blood samples and/or buccal swabs), and were without a diagnosis of dementia. The characteristics of the participants is given in Table 1. The mean age of participants was almost 72 years, and over half were women.

### Early-life abuse/maltreatment

Overall, there were 13% of participants (*n* = 119) who reported that they had been exposed to abuse or maltreatment. The association between this exposure and *BDNF* methylation is shown in Supplementary Figures 1–3 for buccal promoters I and IV, and blood promoter I, respectively. As can be seen, there was little evidence that early life abuse/maltreatment was associated with *BDNF* methylation in later life. The only significant association was a slightly higher buccal *BDNF* methylation at CpG unit 7.8.9 in promoter I in those with childhood abuse/maltreatment. However, after adjustment for age, sex, education, depression, and morbidity, the association was no longer statistically significant (β = 0.07, 95% CI: –0.001 to 0.14, *p* = 0.05). No other association were significant, including blood methylation of the same CpG unit 7.8.9.

### War event or natural disaster

**FIGURE 1:**
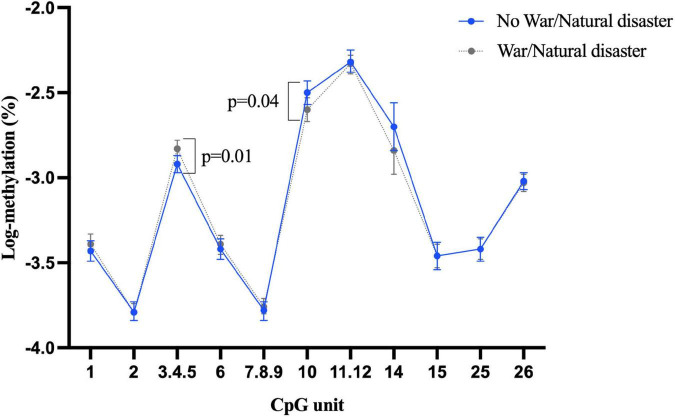
Childhood experience of war/natural disaster and *BDNF* methylation at exon I in blood tissue.

**FIGURE 2:**
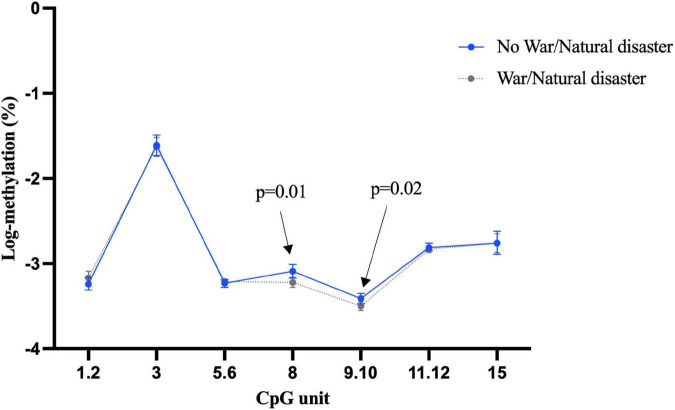
Childhood experience of war/natural disaster and *BDNF* methylation at exon IV in buccal tissue.

Our investigation showed more than half of the participants (*n* = 521) had experienced war or natural disaster in their childhood. There was some evidence that this exposure was associated with differential *BDNF* methylation, even after adjustment for age, sex, education, depression, and morbidity, although the direction of association was not consistent. For example, as shown in Figure 1, individuals who experienced of war/natural disaster had significantly higher blood methylation at CpG unit 3.4.5 in promoter I (β = 0.1, 95% CI: 0.03 to 0.17, *p* = 0.005), but significantly lower methylation at CpG 10 (β = –0.11, 95% CI: –0.21 to –0.01, *p* = 0.04). None of CpG sites in promoter I from buccal tissue were significant (Supplementary Figure 4), but childhood experience of war/natural disaster was associated with a lower *BDNF* methylation in promoter IV at CpG units 8 and 9.10 (CpG 8: β = –0.12, 95% CI: –0.22 to –0.02, *p* = 0.02 and CpG 9.10: β = –0.09, 95% CI: –0.16 to –0.01, *p* = 0.03) (Figure 2).

### Childhood financial difficulties/poverty

**FIGURE 3:**
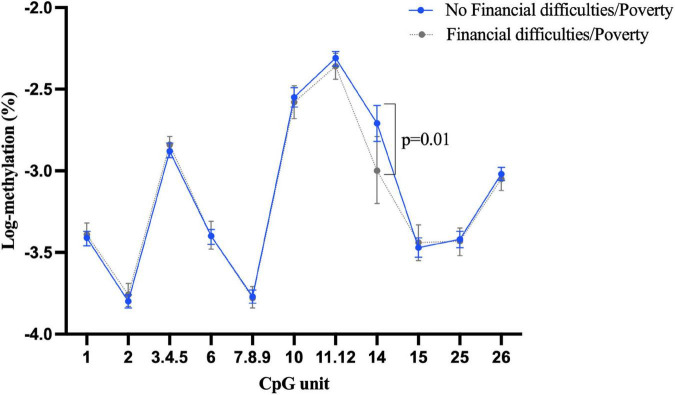
Childhood experience of financial difficulties/poverty and *BDNF* methylation at exon I in blood tissue.

Approximately 22% of participants (*n* = 207) reported they had experienced financial difficulties or poverty in childhood. However, the association between this exposure and *BDNF* methylation was largely null, with no significant difference identified between those with and without childhood financial difficulties/poverty in terms of *BDNF* methylation of both promoter I and IV in buccal tissue (Supplementary Figures 5, 6). The only significant association was a slightly lower blood *BDNF* methylation at CpG 14 in promoter I in those exposed to childhood financial difficulties/poverty (Figure 3) (β = –0.32, 95% CI: –0.55 to –0.09, *p* = 0.005).

## Discussion

Overall, we found no strong evidence for an association between three major childhood adverse experiences and blood and buccal *BDNF* methylation in later life. Childhood abuse/maltreatment was not associated with differential *BDNF* methylation, in either blood or buccal tissue, and financial difficulties/poverty in childhood was only associated with decreased blood methylation at one CpG. The strongest finding was with regards to childhood experience of war/natural disasters, where significant associations were found with *BDNF* methylation, although this was not consistent across gene regions, or tissues, nor was the direction of association consistent (at 3 sites lower methylation was observed with war/natural disaster, but increased methylation at a 4th site). Our significant findings could merely be chance associations, resulting from multiple testing and thus increasing the risk of type 1 error (false positives). Indeed, none of the associations would remain after Bonferroni adjustment based on the number of statistical tests.

One possibility for these largely null findings, is that childhood adverse events have no significant long-term effects on blood or buccal *BDNF* promoter methylation in later life, particularly for those individuals aged 65 years and over. Although findings from some prior rodent and human studies lead to the hypothesis that early-life stress may have a long-lasting effect in altering *BDNF* methylation, which is detectable in peripheral tissue (6, 27, 41–43), none of these studies have measured DNA methylation in older age. Methylation alterations are complex and not fully understood (41). Knowledge about the extent to which peripheral tissues can represent brain functions, and how methylation patterns differ between specific tissues over the life course (29), is still evolving. Further, a growing body of literature suggested the epigenetic patterns are potentially reversible through interventions. For example, mouse models have shown that epigenome-modifying drugs not only have the ability to prevent maltreatment-induced increase of *BDNF* promoter methylation (44), but are also capable of reversing DNA methylation changes resulting from early-life adversity (27, 45–47). Similar findings were reported from human studies, with several studies of depression observing an attenuated *BDNF* methylation change after receiving antidepressant treatment (35, 48–50). Other interventions, such as psychotherapeutic approaches (42) and aerobic exercises (51) also showed potential therapeutic ability in the context of childhood adverse experiences. Taken together, these findings suggest that it might be quite unlikely that DNA methylation changes resulting from early adversity can persist over several decades of life.

*BDNF* is one of the most well-studied genes in response to early life stress. It is highly expressed in the central nervous system and plays a vital role in regulating brain development and facilitating neuron functioning, including proliferation, differentiation, and survival (52–54). Human *BDNF* gene contains eleven functional exons, nine of which are regulated by specific promoter regions (55, 56). Evidence suggests increased methylation at any promoter regions typically leads to decreases in *BDNF* gene expression, as the addition of methyl group inhibits transcription factors to bind properly to regulatory elements (57). Also, stress can suppress *BDNF* expression (58), and decreased *BDNF* expression has been shown to be associated with epigenetic modification of the *BDNF* gene (59), as well as depressive disorder and other mental disorders in late life (22, 60, 61), which eventually leads to our primary interest whether *BDNF* methylation play a role in mediating early life adversities and mental health issues in adults over 65 years of age. However, another possibility for largely insignificant findings is that other genes are involved. For example, *NR3C1*, a stress related gene that codes for glucocorticoid receptors that bind with cortisol or other glucocorticoids and are directly involved in HPA responsivity to stress (62), and some prior studies have reported significant associations between a history of childhood adverse events and *NR3C1* promoter methylation in adulthood (63–66). There is also evidence that other candidate genes, such as interleukin-6 (67) and serotonin transporter gene promoter (68), may be responsive to early-life adverse experiences.

A reason for our largely null findings could also be because individuals with the most severe early-life exposures were not included in our study. The ESPRIT cohort recruited participants who were living in the community with at least 65 years of age, thus those hospitalized or who had died before that age would not have been included. This so-called survival bias may have influenced the findings. Of note, however, prior studies in ESPRIT have demonstrated associations between early-life adversity and later health outcomes, including an increased risk of depression (31) and poorer cognitive performance (33). It has also been shown that some specific events in early life were associated with an increased risk of mortality in older ESPRIT participants, and in particular, war/disaster increased the risk of death for women (32). *Post-hoc* sex-stratified analysis in the current study also found a significant association between war/disaster and decreased *BDNF* methylation in women at two CpG sites of promoter IV in buccal tissue (CpG 8: β = –0.17, 95% CI: –0.31 to –0.04, *p* = 0.01 and CpG 9.10: β = –0.12, 95% CI: –0.22 to –0.02, *p* = 0.02), but not men (CpG 8: β = –0.04, 95% CI: –0.20 to 0.12, *p* = 0.61 and CpG 9.10: β = –0.04, 95% CI: –0.16 to 0.08, *p* = 0.54). The concordance of these associations may be a chance finding, but could also indicate true sex-specific associations. Sex-specific epigenetic changes are a relatively common phenomena in the field, and could be explained by biological differences between the sexes, as well as boys and girls differing in their experience of events. Together these findings would support epigenetic processes as potentially contributing to the long-term effects of early-life adverse events on later-life health, but further work is needed.

There are several limitations to this study, one of the most important to consider is the measure of adversity. Our study only examined the occurrence of these major childhood adverse events, but did not further quantify the severity or exact nature of the adversity. These adverse events would all have differed in intensity and duration, so as the presence of family support, thus the associated stress and impact on the individual would have varied. The likely substantial heterogeneity in exposures may partially account for the overall null associations found here. We didn’t have information either on the level of family support available in childhood, which could help buffer the negative effects of an adverse environment (69–71). Additionally, three major childhood adverse events were specifically selected in this study based on our *prior* hypothesis, however, other types of childhood adversities, such as parental separation or the loss of a family member, should also be carefully considered in future research. Another limitation is the retrospective reporting of early-life adverse experiences. This is likely to lead to some misclassification, considering the long time since its occurrence. However, given that we are measuring an objective outcome in this study, this should not have strongly bias the results in a particular way. Lastly, with the exception of the war/natural disaster exposure in childhood, the two other adverse childhood events were less frequent, especially childhood abuse/maltreatment (reported by < 15%). This meant insufficient power to detect smaller methylation differences, in particular after adjusting for potentially important confounders.

This study also has multiple strengths. Firstly, to authors’ knowledge, this is the first study, focusing on older adults with an age of 65 and over, to examine the relationship between childhood adverse events and *BDNF* promoter methylation. The data collection was embedded in the large longitudinal ESPRIT study, with participants unaware of the objectives of this analysis and childhood adverse experiences obtained in a rigorous manner, which provided high-quality data for this study. Notably, we concentrated on the occurrence of adversities themselves, rather than individuals’ perceptions of their childhood adverse experiences, so that adverse events data was measured objectively. DNA methylation was measured in two tissue types, and for one of these tissues, across two promoter regions of the gene. In addition, a large variety of covariates was controlled in adjustment analysis, including not only the common sociodemographic and lifestyle factors, but also measures of physical and psychological health that were assessed through medical records, trained professionals, standardized interviews, or validated questionnaires.

## Conclusion

There is insufficient evidence of an association between major childhood adverse events, namely abuse/maltreatment, war/natural disaster, and financial difficulties/poverty, and differential *BDNF* promoter methylation in older adults. We suggest future studies to investigate methylation across the genome, rather than focusing on a specific gene, and large, longitudinal studies, with sufficient power to overcome the added risk of false-positive from testing such a number of genes, are preferable. Other epigenetic mechanisms, including histone acetylation and microRNAs, could also be investigated.

## Data availability statement

The original contributions presented in this study are included in the article/Supplementary material, further inquiries can be directed to the corresponding author.

## Ethics statement

The studies involving human participants were reviewed and approved by Ethical Committee of the University Hospital of Kremlin-Bicêtre, France. The participants provided their written informed consent to participate in this study.

## Author contributions

M-LA and KR led the ESPRIT study and the collection of primary data. JR designed the study, undertook the measurement of DNA methylation, and performed the analyses. AZ interpreted the data and wrote the first draft of the manuscript, with supervision from JR. All authors revised the manuscript and approved the final version.

## Footnotes

1
http://www.epidesigner.com/
