# Supplementary material for: Childhood adverse events and BDNF promoter methylation in later-life
Source: Front Psychiatry. 2023 Feb 24;14:1108485. doi: 10.3389/fpsyt.2023.1108485 (PMC9998928; doi:10.3389/fpsyt.2023.1108485)
Supplement: Supplementary file 1 [file Data_Sheet_1.docx]

Supplementary Figure 1. Childhood abuse/maltreatment and *BDNF* methylation at exon I in buccal tissue

Supplementary Figure 2. Childhood abuse/maltreatment and *BDNF* methylation at exon I in blood tissue

Supplementary Figure 3. Childhood abuse/maltreatment and *BDNF* methylation at exon IV in buccal tissue

Supplementary Figure 4. Childhood experience of war/natural disaster and *BDNF* methylation at exon I in buccal tissue

Supplementary Figure 5. Childhood experience of financial difficulties/poverty and *BDNF* methylation at exon I in buccal tissue

Supplementary Figure 6. Childhood experience of financial difficulties/poverty and *BDNF* methylation at exon IV in buccal tissue
